# Eggshell composition and surface properties of avian brood-parasitic species compared with non-parasitic species

**DOI:** 10.1098/rsos.221023

**Published:** 2023-05-24

**Authors:** Stephanie C. McClelland, Marie R. G. Attard, James Bowen, Nicholas P. C. Horrocks, Gabriel A. Jamie, Tanmay Dixit, Claire N. Spottiswoode, Steven J. Portugal

**Affiliations:** ^1^ Department of Biological Sciences, School of Life and Environmental Sciences, Royal Holloway University of London, Egham, Surrey TW20 0EX, UK; ^2^ School of Engineering and Innovation, Open University, Milton Keynes MK7 6AA, UK; ^3^ Department of Zoology, University of Cambridge, Downing Street, Cambridge CB2 3EJ, UK; ^4^ Cambridge Institute of Therapeutic Immunology and Infectious Disease (CITIID), Jeffrey Cheah Biomedical Centre, Cambridge Biomedical Campus, Cambridge University, Cambridge CB2 0AW, UK; ^5^ FitzPatrick Institute of African Ornithology, DST-NRF Centre of Excellence, University of Cape Town, Rondebosch 7701, Cape Town, South Africa; ^6^ The Natural History Museum, Tring, Herts HP23 6AP, UK

**Keywords:** brood parasitism, calcium carbonate, coevolutionary arms race, cuckoos, profilometry, surface topography

## Abstract

The eggs of avian obligate brood-parasitic species have multiple adaptations to deceive hosts and optimize development in host nests. While the structure and composition of the eggshell in all birds is essential for embryo growth and protection from external threats, parasitic eggs may face specific challenges such as high microbial loads, rapid laying and ejection by the host parents. We set out to assess whether eggshells of avian brood-parasitic species have either (i) specialized structural properties, to meet the demands of a brood-parasitic strategy or (ii) similar structural properties to eggs of their hosts, due to the similar nest environment. We measured the surface topography (roughness), wettability (how well surfaces repel water) and calcium content of eggshells of a phylogenetically and geographically diverse range of brood-parasitic species (representing four of the seven independent lineages of avian brood-parasitic species), their hosts and close relatives of the parasites. These components of the eggshell structure have been demonstrated previously to influence such factors as the risk of microbial infection and overall shell strength. Within a phylogenetically controlled framework, we found no overall significant differences in eggshell roughness, wettability and calcium content between (i) parasitic and non-parasitic species, or (ii) parasitic species and their hosts. Both the wettability and calcium content of the eggs from brood-parasitic species were not more similar to those of their hosts' eggs than expected by chance. By contrast, the mean surface roughness of the eggs of brood-parasitic species was more similar to that of their hosts’ eggs than expected by chance, suggesting brood-parasitic species may have evolved to lay eggs that match the host nest environment for this trait. The lack of significant overall differences between parasitic and non-parasitic species, including hosts, in the traits we measured, suggests that phylogenetic signal, as well as general adaptations to the nest environment and for embryo development, outweigh any influence of a parasitic lifestyle on these eggshell properties.

## 1. Introduction

All birds lay eggs with calcified shells, which provide essential functions for embryo health and development [1,2]. The shell protects the embryo from mechanical, solar and microbial damage [3–5], modulates gas exchange with the environment [6,7] and provides calcium to the embryo for tissue development [8–10]. The microstructure and outer topography of the eggshell is a highly labile trait that varies across avian lineages to meet specific demands of the embryo, based on their nest environment and parental incubation strategy [5,11,12]. For example, eggs of species that typically nest in damp and humid environments exhibit higher rates of gas exchange under standard conditions than those of species which nest in drier environments [6] and have cuticular nanospheres present on the outer surface of the eggshell [12]. These cuticular nanospheres prevent the accumulation of water on the eggshell surface, helping protect the egg from bacterial and microbial infection. This suggests that eggshell properties have evolved to reflect the specific challenges posed by the environment in which the egg is laid [13].

For the approximately 1% of extant birds that are obligate brood-parasitic species [14], eggshell properties may be particularly important for determining embryonic survival. While only a small proportion of birds are brood-parasitic, the number of species affected by the presence of these parasites is far greater. Since brood-parasitic species do not raise their own young and instead deposit their eggs in the nests of host species [15–17], parasitic parents have no subsequent impact on the development or external conditions experienced by their eggs after laying. Therefore, producing a suitably adapted eggshell for survival in a host's nest should be under strong selection [18,19]. Previous studies have demonstrated that brood-parasitic species typically have thicker eggshells than their hosts, with increased microhardness to protect against puncturing during host ejection [20,21] or breakage during rapid laying [22]. Eggs of brood-parasitic species also exhibit low water vapour conductance across the eggshell, which may increase the aerobic fitness of parasitic hatchlings [23,24]. Less attention has been given to other mechanical and structural adaptations of parasitic eggs, which, if the host does not reject the egg, may aid the embryo's survival to hatching.

Along with protecting the embryo from pathogens, the composition of the shell is important for limiting physical damage. The quantity and structure (including the geometry and orientation) of calcium carbonate (CaCO_3_) particles that form the bulk of the shell (approx. 85–99%) have a significant impact on the mechanical properties of the eggshell [25,26]. However, calcium can be difficult to source for many bird species [27], and so their investment of calcium in eggshell formation must weigh this cost against the requirements of the egg [10]. Brood-parasitic species typically lay many more eggs than non-parasitic species [14,28]. Moreover, the eggs of brood-parasitic species are thicker for their size than those of non-parasitic species [20,21], with the increased thickness potentially being achieved through a higher CaCO_3_ content. The greater thickness is generally thought to be a defence against potential eggshell puncturing by hosts, and/or to provide an element of protection during the rapid laying process frequently associated with some brood-parasitic species [14]. It could, therefore, be hypothesized that eggshell CaCO_3_ requirements would be greater for brood-parasitic species. A large-scale macroecological study of 222 bird species, however, found that there is no interspecific correlation between eggshell CaCO_3_ content and eggshell thickness [10], suggesting that thickness in avian eggshells is achieved potentially through other mechanisms besides simply having a greater CaCO_3_ content. Nevertheless, the rapid embryonic development of brood-parasitic species and their shorter incubation time [14,29] could result in greater CaCO_3_ eggshell content being required, as the shell provides the main supply of calcium for embryonic bone and muscle development [30]. Overall, therefore, parasitic eggs might be hypothesized to contain more CaCO_3_ than non-parasitic eggs, potentially as a result of their thicker shells or because the development of parasite chicks places greater demands on calcium. Conversely, the eggs of brood-parasitic species might be expected to exhibit lower CaCO_3_ content per egg than those of other species, since the greater number of eggs laid by brood-parasitic females could mean that each egg contains a smaller proportion of a limited supply of CaCO_3_ than would otherwise be the case.

A particular challenge for the eggs of brood-parasitic species is the risk of infection. It has been shown in at least two brood-parasitic systems that both host and parasitic eggs in parasitized nests have a higher microbial load and greater risk of trans-pore infection than do the eggs of host eggs in non-parasitized nests [31,32]. Higher microbial loads in parasitized nests are attributed to synergistic effects of microbes introduced by the laying parasite, those already present in the host nest's microbiome and, in some parasitic systems, from the decomposition of host eggs damaged or broken by the parasite during laying [31,33,34]. However, the eggshells of brood-parasitic species need not necessarily experience greater bacterial loads than host eggshells; for instance, the eggs of brood-parasitic great spotted cuckoos (*Clamator glandarius*) had a lower density and quantity of microbes on their eggshell surface than those of their hosts, Eurasian magpies (*Pica pica*), in the same nest [31]. This difference in bacterial load between host and parasitic eggs suggests that parasitic eggshells could possess specialized surface properties to reduce microbial adhesion [35,36].

Whether surface structures of brood-parasitic eggs have evolved to minimize microbial infection has not been investigated. However, eggs under particularly high risk of infection, such as those of compost-nesting malleefowls (*Leipoa ocellata*), have evolved extremely hydrophobic (water-repellent) eggshell surfaces comprising nanosphere-type structures [36,37]. A less wettable surface causes water to ‘bead up’ rather than spread out over the shell surface, and in doing so, the water traps harmful microbes in these droplets and minimizes the formation of biofilms [5]. Thus, surfaces with low wettability (contact angle (CA), the degree to which eggshell surfaces can repel or attract water) [38] can influence the exchange of respiratory gases and water vapour through the eggshell pores, by preventing the pores from being blocked by water or dirt. In eggshells, the curvature of the egg can additionally influence wettability, with species with smaller and thus more curved eggs tending to have eggshell surfaces that are more hydrophobic [38]. A less well-understood aspect of eggshell surface dynamics is surface roughness (S_a_, the deviation of a surface from its mean plane, characterized by variance in height, forming peaks and valleys) [38]. It had been assumed that eggshell CA may be governed by the S_a_ of the surface [1,39], but on other natural surfaces, CA has been shown to be determined not only by S_a_ (the Wenzel principle, e.g. Kubiak *et al.* [40]) but also by the chemical characteristics of the surface itself. It has not been determined how S_a_ interacts with CA and other physical factors for eggshells, but we might hypothesize that the eggs of brood-parasitic species exhibit greater S_a_ than non-parasitic species to decrease wettability and so assist in protecting against possible microbial infection.

Finally, for parasitic host specialists (i.e. those that parasitize a single host species, unlike parasitic host-generalists, which can parasitize multiple species), it could be hypothesized that eggshell properties would match those of their hosts due to adaptations to a shared nest environment. This could also be true for some parasitic species often considered to be host-generalists (e.g. common cuckoos (*Cuculus canorus*) or cuckoo finches (*Anomalospiza imberbis*), in which maternally inherited host specialization can nonetheless exist at the level of host-races or gentes (singular gens; i.e. one or more lineages of females favouring, and typically mimicking, a specific host species' nest) within a species [41,42]. Maternal inheritance provides a mechanism for within-species specialization not only in parasitic egg mimicry but also in other traits expressed only by females, such as egg structural traits. Eggshell thickness, for example, differs between common cuckoo gentes, relative to the likelihood of rejection from their hosts [43]; the greater the probability of a host species rejecting a parasitic egg, the thicker the eggshell of the corresponding cuckoo gens. In a similar way, adaptations to the immediate nest environment could be shared between each gens within a species and its respective host, as well as between host-specialist brood-parasitic species and their respective hosts.

In this study, we first test the hypothesis that eggs of brood-parasitic species are *different* to non-parasitic bird species, including hosts and non-hosts, and exhibit specific adaptations to a parasitic lifestyle with respect to their surface properties (S_a_ and CA) and CaCO_3_ content. Specifically, this hypothesis predicts that relative to the eggs of their respective hosts, the eggs of parasitic species should (i) exhibit greater S_a_ and lower CA, to defend against the increased risk of infection previously recorded in parasitized nests, and (ii) have *either* proportionally *greater* CaCO_3_ content of the eggshell, compared with hosts, to support rapid bone development and, potentially for certain brood-parasitic species, counter against host puncture ejection *or* proportionally *less* CaCO_3_ due to the quantity of eggs laid. Such adaptations would function to provide brood-parasitic species with a competitive edge within the host nest environment. Second, we test the alternative hypothesis that the eggs of host specialist brood-parasitic species (at the individual or matriline level) *match* those of their hosts in terms of S_a_ and CA, due to adaptations to the local nest environment, and are, therefore, more similar to their hosts than to other non-parasitic bird species. These two hypotheses correspond, respectively, to whether either a parasitic lifestyle or the local host nest environment primarily drives eggshell characteristics in avian brood-parasitic species.

## 2. Material and methods

### 2.1. Overview of samples, sources and preparation

Eggshell surface roughness (S_a_), wettability (CA) and CaCO_3_ content were measured in eggshells from brood-parasitic species (*N* = 14; covering five of the seven independent transitions to obligate brood parasitism [44]) and non-brood-parasitic species (*N* = 39) (see electronic supplementary material, table S1 for a full list of sources and sample sizes). The eggshells of species which were neither brood-parasitic nor known hosts of brood-parasitic species were measured as controls, to assess the general phylogenetic spread of S_a_, CA and CaCO_3_ eggshell content. All eggs were from different clutches. Measurements were taken from either whole eggs or eggshell fragments which were sourced from the field or museums (see electronic supplementary material). Sample sizes for species are not identical for S_a_, CA and CaCO_3_ due to some eggs not being available for potentially destructive analyses.

Most eggshells (total *N* = 104) were obtained from the destructive collection (Class II) of the Natural History Museum collection at Tring (UK), where minimal clutch information was available [45]. Many of these specimens comprised fragments only, with no information on which eggshell region they represent. Eggshell fragments (*N* = 37) from the Western Foundation of Vertebrate Zoology in Camarillo (California, USA) were sampled from the equatorial region of the egg. Equatorial fragments were also sampled for all eggs collected during fieldwork in the Choma region, Zambia (*N* = 136) (collection site information in McClelland *et al.* [24] and Spottiswoode & Stevens [46]). Samples were first used to measure surface S_a_, followed by CA, to ensure that water droplets did not interfere with surface roughness measures. Calcium carbonate content was determined afterwards since this procedure destroys the sample.

### 2.2. Profilometry measurements of surface roughness

**Figure 1.**
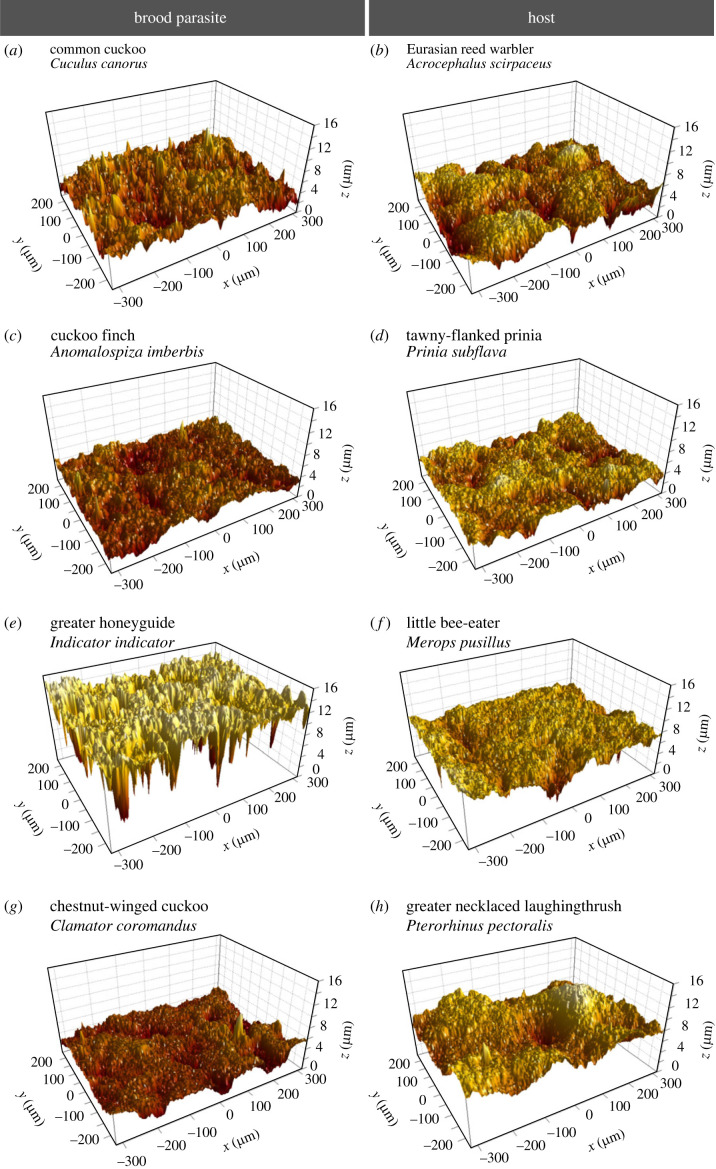
Surface topography and roughness of parasitic and host eggshells. The colour gradient distinguishes between higher surface points (light yellow) and deeper points (darker brown)*.* Images are of brood-parasitic species (left column) and respective host (right column) pairs: (*a*) common cuckoo (*C. canorus*), (*b*) Eurasian reed warbler (*A. scirpaceus*), (*c*) cuckoo finch (*A. imberbis*), (*d*) tawny-flanked prinia (*Prinia subflava*), (*e*) greater honeyguide (*Indicator indicator*), (*f*) little bee-eater (*M. pusillus*), (*g*) chestnut-winged cuckoo (*C. coromandus*), (*h*) greater necklaced laughingthrush (*P. pectoralis*). Of the fragments shown, the greater honeyguide had the greatest surface roughness (2384 nm S_a_), and the cuckoo finch had the lowest surface roughness (745 nm S_a_), with the overall order, in decreasing order of roughness, being (*e*), (*a*), (*h*), (*b*), (*d*), (*f*), (*g*) and (*c*). Scans were generated by SPIP software (Image Metrology, Denmark).

Eggshell surface roughness (S_a_) was measured for 283 eggshells from 45 bird species, including 125 eggs from 14 parasitic species. Shell fragments were placed on a glass slide with the outer shell surface facing upwards. For stability, Blu Tack (Bostik Ltd, Leicester, UK) was used to secure the fragment in position from underneath, without touching the outer shell surface. Whole eggs were balanced on the inverted lid of a Falcon tube for stability when under the microscope, with the longitudinal axis of the egg parallel to the bench. Eggshell samples were placed under a non-contact optical profilometer using a white light source interferometric microscope (Leica DCM 3D, Germany). We used an objective magnification of 20× to give a pixel resolution of 768 × 576 (measurable area 636 × 477 µm). Three to four non-overlapping locations on the fragment were selected and measured at 100 focal planes (totalling a vertical depth of 100 µm from the outermost surface). Two-dimensional and three-dimensional digital elevation models of each shell location were saved as separate image files (figure 1).

SPIP (Scanning Probe Image Processor, SPIP v. 4.4.3.0, Image Metrology, Hørsholm, Denmark) was used to process images and extract measurements of surface roughness, S_a_, which was calculated in units of nanometres (nm) from an average of images taken at four locations per egg. S_a_ represents the arithmetic mean distance of each point from the mean focal plane of the sample. Due to the curved surface of the fragments, a second-order polynomial function was applied to the plane correction [47]. Digital elevation models were created in two dimensions and three dimensions for each scan.

### 2.3. Wettability (contact angle) measurements

Surface CA was measured from 148 eggshells across 39 bird species, including 47 eggs from 11 parasitic species. The wettability of the eggshell surface was determined by measuring the CA of a sessile water droplet on the shell surface (hydrophilic = CA < 90°, hydrophobic = CA ≥ 90° < 150° and superhydrophobic = CA ≥ 150°) using a DSA 100 Drop Shape Analyzer (Krüss, Germany). Protocols were based on D'Alba *et al*. [36]. The shell fragment was placed on a slide mounted on a stage in front of the analyser's highspeed camera, and a 4–9 µl drop of deionized water was deposited onto the flattest plane surface of the shell using a flat-pointed needle tip. The volume of the drop was estimated by Advance software (Krüss ADVANCE 1.8-01, Germany) from the images recorded. The droplet was placed on the surface of the eggshell at room temperature by lifting the stage until the droplet on the flat-pointed needle tip contacted and adhered to the shell surface. The stage was then gently lowered away from the syringe. This method prevented the spreading of the droplet that would be caused by the force of dropping from the syringe point onto the fragment surface. As droplet spreading was minimal after 5 s, we recorded the CA between the droplet and eggshell surface on both the left- and right-hand side every second for the first 5 s once the droplet was no longer in contact with the syringe. Our analysis is based on average CA recorded at 5 s, by which time the water droplet had settled. The Advance software used a Young–Laplace model to apply a curved baseline to account for the curvature of the eggshell surface when measuring CA [38].

### 2.4. Calcium carbonate content of brood-parasitic and other species

To determine eggshell CaCO_3_ content, eggshells were ‘ashed’ in a muffle furnace (AAF 1100; Carbolite, Hope, UK) following procedures in McClelland *et al*. [10]. Shell fragments were placed in 10 ml ceramic crucibles and weighed in grams to four decimal places on a precision balance (Sartorius 1265 MS, Gottingen, Germany). Empty crucible mass was recorded beforehand. Eggshell fragments were then dried to a constant mass by being placed in an oven at 60°C for 24–32 h. Shell fragments were weighed twice daily, between 09.00–10.00 and 16.00–17.00, until no change in mass was detected for two consecutive weighing sessions, at which point they were considered ‘dry’. The crucibles containing the dry shell fragments were then placed into the muffle furnace for 18 h at 500°C to burn off the organic component of the shell. The crucible with the shell ash was placed in a glass desiccator immediately after removal from the furnace to cool down before being weighed again. The mass of the empty crucible was deducted to calculate the dry masses of the shell fragment and ash. Calcium carbonate content was calculated as the ash mass of the shell fragment, as a percentage of the dry mass of the shell fragment. Other inorganic minerals in the eggshell were not considered separately from CaCO_3_ as they occur in extremely small quantities (e.g. less than 0.1% of the eggshell comprises phosphorus and magnesium) [48,49].

### 2.5. Statistical analyses

All statistical analyses were conducted in R statistical software v. 3.6.3 [50] using the front-end ‘R Studio’ [51]. Figures were produced using the package ‘ggplot2’ [52]. We constructed two separate phylogenetically informed mixed models (PMM) to test whether parasitic species differed from non-parasitic species in either their eggshell S_a_ or their CA using the R package ‘sommer’ [53]. This approach accounts for phylogeny, which is necessary as species are not statistically independent due to shared ancestry [54,55]. A phylogenetic tree of all species included in this study can be found in the electronic supplementary material, taken via the R package ROTL [56]. We estimated the importance of species' shared evolutionary history using heritability (*H*^2^), a measure of phylogenetic signal ranging between 0 and 1, that we calculated from the estimated phylogenetic variance in the PMM models [57]. The interpretation of *H*^2^ is identical to that of Pagel's *λ* in phylogenetic generalized least-squares models [58]. *H*^2^ = 0 indicates no phylogenetic signal and *H*^2^ = 1 indicates that phylogeny fully explains any observed patterns, meaning that trait variation between species is consistent with Brownian motion evolution.

A PMM was constructed with S_a_ as a response variable and parasitic status (parasitic or non-parasitic) as a predictor variable. The phylogenetic model accounts for species variance. A second PMM was used to compare CA across all eggs measured, using parasitic status (parasitic or non-parasitic) as the predictor variable. A single CA was used per egg, as this measure is highly repeatable at adjacent locations on the same egg [38], so egg identity was not controlled for in this model. As *p-*values are not generated as standard with this method, the *p-*values of predictors in PMM models were calculated from a χ^2^-test to compare the model containing the predictor and the same model without that predictor. A linear mixed model was used to test whether eggshell S_a_ was a good predictor of CA, based on the 67 eggs from 19 species (including host and parasitic eggs) where both values could be obtained from the same eggshell fragment. Linear mixed models were performed using the R packages lme4 [59] and lmertest [60], with species included as a random effect. The difference in eggshell CaCO_3_ content between brood-parasitic species and non-parasitic species was tested using the same statistical methods as S_a_ and CA, as described above.

Permutation procedures [61] were used to determine whether brood-parasitic species were more similar to their host in eggshell surface properties than would be expected by chance. These compared the differences in mean S_a_ and CA between either an egg of a brood-parasitic species and an egg of their host species, or between a brood-parasitic egg and a randomly selected egg of a non-parasitic species. To compare mean S_a_, the mean per egg was calculated and 121 pairwise comparisons were constructed of different egg combinations: either parasite–host (*N* = 52) or parasitic–random (*N* = 69). For CA, 56 pairwise comparisons of egg combinations were constructed: either parasite–host (*N* = 33) or parasite–random (*N* = 23).

Four species in our dataset (both honeyguide species, common cuckoos and cuckoo finches) parasitize multiple host species. For both honeyguide species and cuckoo finches, our host samples came from the same host species as the parasitic eggs we sampled (i.e. the host sample matched parasitic ‘gens’). We only sampled one host species per parasitic species, so it was not possible to test for any between-gens differentiation within parasitic species, despite the theoretical expectation that such differences could evolve (see Introduction). For common cuckoos, however, this was not the case, as the eggs we sampled from the destructive collection at Tring did not all have host nest information accompanying them, though all host nests were one of either Eurasian reed warbler (*Acrocephalus scirpaceus*), great reed warbler (*Acrocephalus arundinaceus*) or meadow pipit (*Anthus pratensis*). Thus, we used the mean value for these three hosts as a measure of S_a_ and CA of hosts of common cuckoos. *Vidua* finches (*Vidua macroura*, *Vidua obtusa*, *Vidua purpurascens*) were compared with their specific hosts collected from the field (Zambia), while museum specimens of chestnut-winged cuckoos (*Clamator coromandus*) were compared with their primary hosts, greater necklaced laughingthrushes (*Pterorhinus pectoralis*) [62].

## 3. Results

### 3.1. Do the eggshell surface properties of brood-parasitic eggs differ from non-parasitic eggs?

**Figure 2.**
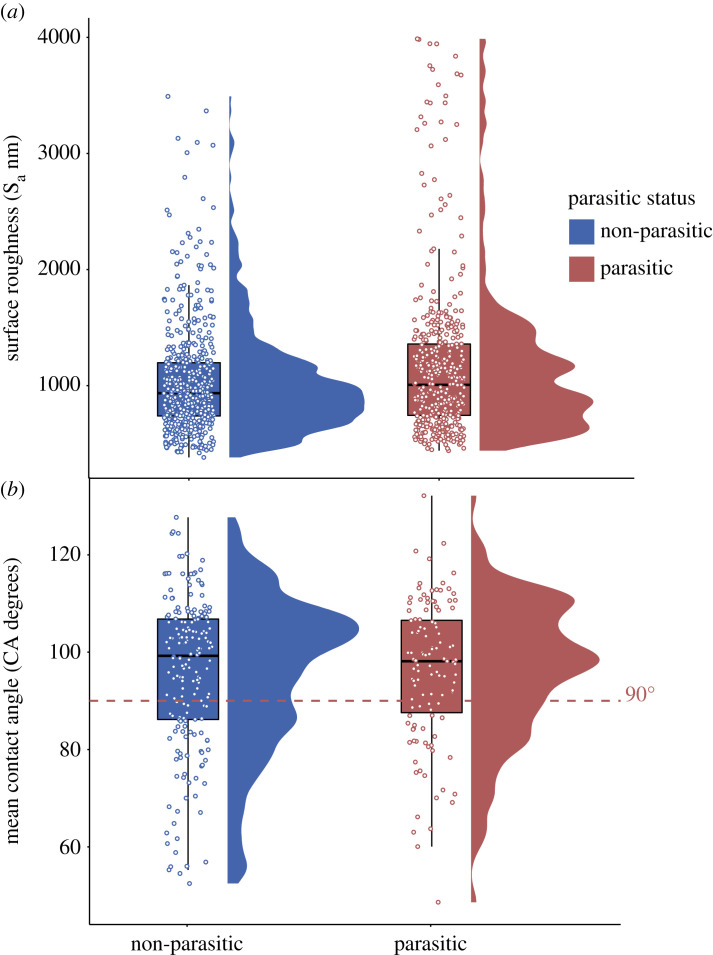
(*a*) Avian eggshell surface roughness (S_a_ nm) of non-parasitic species (left, blue) and brood-parasitic species (right, red). (*b*) Avian eggshell surface wettability (CA), as measured by the mean CA of a water droplet on eggshell surfaces of non-parasitic species (left, blue) and brood-parasitic species (right, red). The red dotted line at 90° is the threshold between hydrophilic (less than 90°) and hydrophobic (greater than or equal to 90°) eggshell surfaces. No significant differences were evident between the eggs of brood-parasitic and non-parasitic eggs for either trait (S_a_: *t* = 0.09, *p* = 0.26; CA: *t* = 0.59, *p* = 0.79). Boxplots show the interquartile range and median value as a line, with whiskers encompassing values within 1.5 times the interquartile value. The distribution of the data is shown in scatterplots (overlaid) and frequency plots (alongside each pairing). Each data point shows a measurement from one egg, and each egg is from a different nest; analyses take into account phylogenetic non-independence, as well as non-independence of multiple eggs per species (see Methods).

The roughness (S_a_) of eggshell surfaces ranged from 439 to 3987 nm in the eggs of brood-parasitic species and 381–3491 nm in non-parasitic eggs (figures 1 and 2). S_a_ of the eggs of brood-parasitic species was not significantly different from eggs of non-parasitic species in general (PMM, estimate = 26.82, s.e. ± 275.0, *t* = 0.09, *p* = 0.26, figure 2). There was a strong phylogenetic signal in the measurements of S_a_ (*H*^2^ = 0.95 ± 0.01 s.e.), suggesting that phylogenetic relatedness explains much of the variation in S_a_. There was also no significant difference in eggshell surface wettability (CA) between brood-parasitic species and non-parasitic eggs (PMM, estimate = 1.537, s.e. ± 2.61, *t* = 0.59, *p* = 0.79, figure 2). The eggs of most species had eggshell surface CA of less than 90° and so were categorized as hydrophobic (figure 2). The phylogenetic signal for CA was low (*H*^2^ = 0.03 ± 0.07), suggesting that life-history or other selective pressures influence variation in eggshell CA across species to a greater extent than phylogenetic relatedness. S_a_ was not correlated with CA (for all eggs measured) based on Pearson's correlation coefficient (*R*^2^ = −0.02, 95% CI [−0.35, 0.13]), and nor was S_a_ a good predictor of CA (linear mixed model (LMM), estimate = −0.0006, s.e. = 0.003, *t*_35.5_ = −0.197, *p* = 0.845). The correlation between mean S_a_ and CA was low for both the eggs of brood-parasitic species (*R*^2^ = −0.07, 95% CI [−0.41, 0.28]) and non-parasitic eggs (*R*^2^ = −0.003, 95% CI [−0.31, 0.37]).

### 3.2. Does the nest environment determine eggshell characteristics? Comparison of mean surface roughness and mean contact angle between brood-parasitic species and their hosts

**Figure 3.**
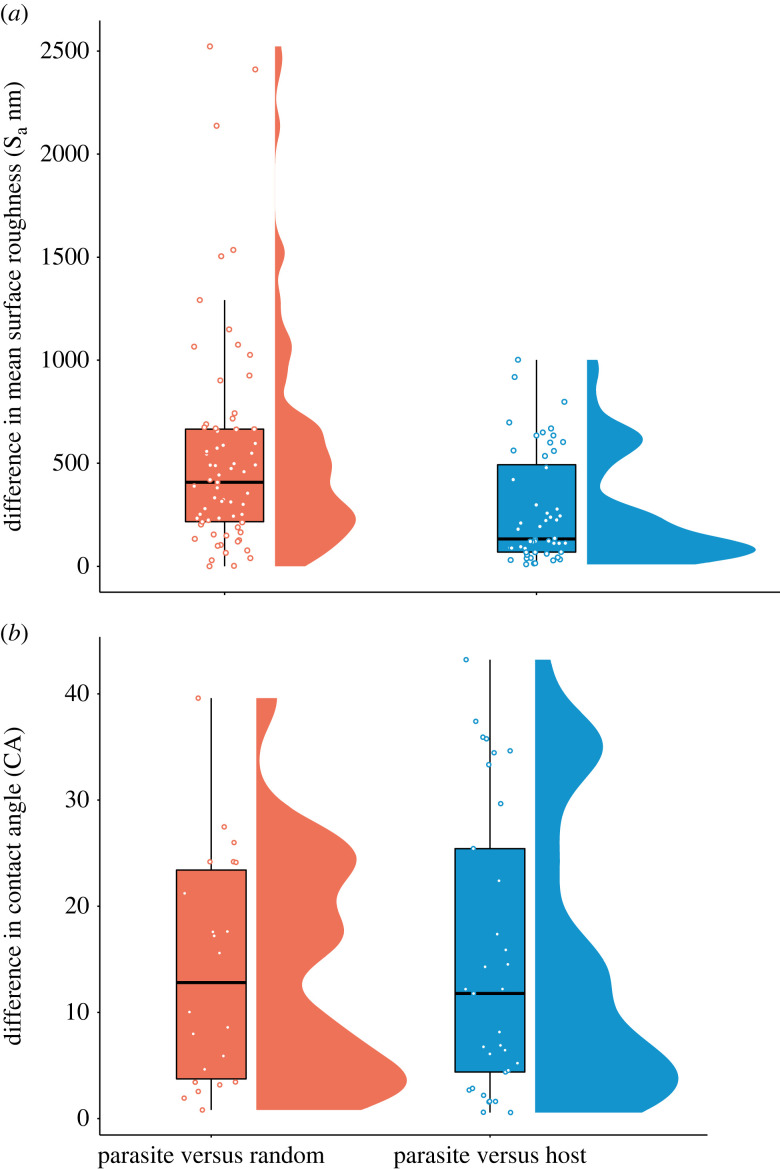
The difference in avian eggshell surface roughness (S_a_) and CA between the eggs of parasitic, hosts and non-host species. (*a*) There was a significantly smaller difference in mean S_a_ between brood-parasitic eggs and their hosts' eggs than between brood-parasitic eggs and randomly allocated non-parasitic eggs (*t*_118_ = 3.17, *p* = 0.002). (*b*) The difference in CA of sessile water droplets between brood-parasitic eggs and their hosts’ eggs was not significantly different from that between brood parasites and randomly allocated non-host eggs (*t*_48_ = 0.10, *p* = 0.92). Boxplots show the interquartile range and median value as a line, with whiskers encompassing values within 1.5 times the interquartile value. The distribution of data is shown in scatterplots (overlaid) and frequency plots (alongside each pairing). Each data point shows a measurement from one egg, and each egg is from a different nest.

The mean S_a_ of the eggs of brood-parasitic species was more similar to the mean S_a_ of their hosts' eggs than the parasitic eggs were to random non-parasitic species eggs (estimate = 247.52, s.e. ± 77.98, *t*_118_ = 3.17, *p* = 0.002, figure 3). By contrast, the mean CA of brood-parasitic eggs were no more similar to the mean CA of their hosts eggs than the parasitic eggs were to a random species egg (estimate = 0.34, s.e. ± 3.60, *t*_48_ = 0.10, *p* = 0.92, figure 3), indicating that eggshell CA does not differ depending on parasitic status.

### 3.3. Calcium carbonate content of brood-parasitic species versus non-parasitic species

**Figure 4.**
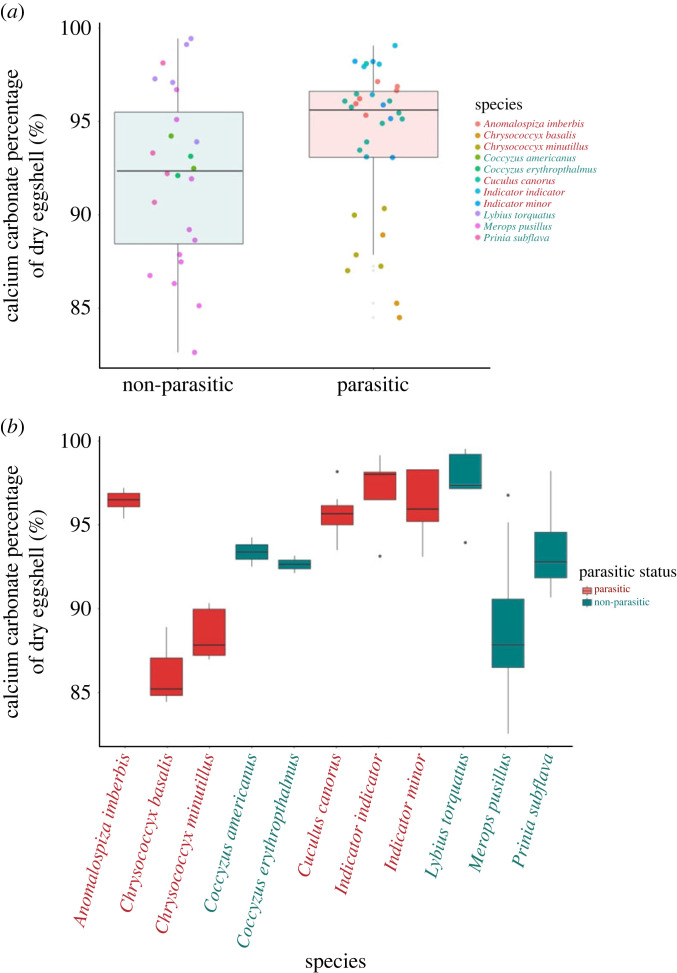
(*a*) Eggshell CaCO_3_ content of brood-parasitic species compared with those of non-parasitic species. Boxplots illustrate the group mean and interquartile range and whiskers cover 1.5 times the interquartile range. Eggs from different species are colour coded on the scatterplot (red text denotes parasitic species); datapoints are species means. (*b*) Eggshell CaCO_3_ range for each species sampled. Grey dots denote points that are outside the 1.5 interquartile range.

After accounting for phylogenetic relatedness, the eggshell CaCO_3_ content of brood-parasitic species was not significantly different from that of non-parasitic species (estimate = 0.013, s.e. ± 0.02, *t* = 0.5369, *p* = 0.62, figure 4). The phylogenetic signal for CaCO_3_ content (*H*^2^ = 0.88 ± 0.06) suggests that while a strong phylogenetic signal is retained in CaCO_3_, the trait has evolved according to a process in which the effect of phylogeny is nonetheless weaker than in the Brownian model.

## 4. Discussion

Coevolution between brood-parasitic species and their hosts has resulted in selection of a wide variety of adaptations related to their eggs [43,63]. Here, we found that eggshells of brood-parasitic species show no distinct *overall* differences in their roughness (S_a_), wettability (CA) or CaCO_3_ content compared with their close relatives or their hosts, refuting our first hypothesis that eggshell structures of brood-parasitic species exhibit specific adaptations to a parasitic lifestyle, which host eggshells lack. However, the mean surface S_a_ of the eggs of brood-parasitic species was more similar to the mean surface S_a_ of their hosts' eggs, than to the eggs of random non-parasitic species in our dataset. This similarity in S_a_ between parasites and hosts supports the alternative hypothesis that a shared nest environment is likely to influence S_a_.

### 4.1. Comparisons of eggshell roughness between brood-parasitic and host species

We hypothesized that the eggshells of brood-parasitic species would exhibit greater surface S_a_ to defend against the increased risk of infection previously recorded in parasitized nests [31,32], e.g. due to reduced bacterial adhesion via low CA. We did not find an overall significant difference in the eggshell S_a_ between parasitic eggs compared with that of non-parasitic species. Thus, a parasitic lifestyle does not appear to have resulted in the evolution of eggs with consistently higher surface S_a_. Instead, the S_a_ of brood-parasitic eggs was more similar to the S_a_ of their hosts' eggs than to the eggs of random species in our dataset. The phylogenetic signal for S_a_ was high (*H^2^* = 0.95), implying that phylogenetic relatedness explains much of the variation in this eggshell trait; nonetheless, the small but significant element of convergence in S_a_ between hosts and parasites is suggestive of convergent adaptation to a shared nest environment. A degree of convergence is especially striking given the wide phylogenetic differences between some of the brood-parasitic species and their hosts (e.g. common cuckoos and greater honeyguides parasitize species from different orders).

Why was the phylogenetic signal in S_a_ so high? Our study focuses on brood-parasitic species and their hosts, and all hosts in the present study bar two (black-collared barbets *Lybius torquatus* and little bee-eaters *Merops pusillus*) are passerines. Many of the passerine host species in this study have comparable cup nest types, and thus S_a_ could be adapted for that nest design. The nest environment is likely to exert strong selection pressure on S_a_, as has been documented in other eggshell characteristics, such as conductance [3,6,7]. For example, crudely taking mean eggshell roughness for cavity-nesting parasites (1765 ± 900 nm) versus all other nest types (1056 ± 307 nm) suggests that cavity nesters have higher eggshell S_a_, potentially to contend with the more humid conditions of the nest environment, and/or the higher microbial load from the parasites puncturing the host eggs (e.g. greater honeyguides). However, this should be interpreted with caution, as interspecific eggshell variation is high within each of these crude categories (cavity nesters versus all other nest types), and such a pattern in roughness may just reflect a phylogenetic artefact due to both cavity-nesting species being honeyguides (*Indicatoridae*) that also, at least sometimes, puncture host eggs. Overall, eggshell S_a_ appears to be more adapted to the immediate nest environment, rather than there being significant differences between parasitic species and their hosts.

Eggshell maculation has previously been demonstrated to exhibit photodynamic antimicrobial activity [64,65]. Protoporphyrin, in particular, reduces the survival of gram-positive bacteria to only 0.01%, under lit conditions, but has no impact on gram-negative bacteria. Infections from bacteria, penetrating the shell via the pores, are a significant mortality risk to the developing embryo, particularly before incubation begins [66]. Pigmentation on the outer surface can also alter the general topography of the shell. In some species, pigment spots are evident on the surface due to the obvious change in structure, whereas in other species, there is no apparent change in the surface structure associated with the pigment spot [67]. Thus, whether pigmentation consistently has different surface properties is not currently known and worthy of further investigation. The findings of such a study would have substantial implications for understanding our observation that the S_a_ of brood-parasitic eggs was more similar to the S_a_ of their hosts’ eggs than to the eggs of a random species. For those brood-parasitic species which exhibit eggshell mimicry, it is possible that the requirements for the brood parasite to match the maculation colour and pattern of host eggs result in similar S_a_ properties.

We expected that only parasitic eggs would demonstrate adaptations to the increased risk of microbial infection in parasitized nests. We did not expect the same in their hosts because of the inconsistent nature of any selective pressures acting on the potential hosts. The presence of a parasitic egg may change the bacterial microbiota in the host nest. In one host–parasitic system, fresh common cuckoo eggs and incubated host eggs in unparasitized nests (where no parasite or parasitic egg was present) were the most dissimilar in the microbiome, compared with cuckoo eggs from different nests, host eggs from different parasitized nests, and host eggs from different unparasatized nests [32]. Any parasitic egg is, therefore, theoretically always being laid into a nest with a higher risk of microbial infection; however, not every host egg will experience these conditions, since many host nests are not parasitized. Those host nests which are parasitized will probably fail to fledge chicks for other reasons (i.e. parasitic chicks ejecting or otherwise killing host chicks) and so will never be exposed to selection. Thus, we suggest that host eggs will not necessarily show eggshell adaptations to contend with the higher risk of microbial infection arising from parasitism.

A limitation of our study is its relatively small sample sizes and the broad measurements and categorizations of S_a_. It is unclear whether the range in S_a_ observed in this study is sufficiently large to influence the effectiveness of the shell in aiding against microbial infection. We assumed that a higher S_a_ would result in better defence against microbial infection, but studies focusing on artificial surfaces have actually shown a nonlinear relationship between the two: high surface S_a_ can actually increase the surface area available for bacterial attachment, while additionally providing a platform for adhesion [68,69], until a certain S_a_ threshold where further increases in S_a_ can then reduce bacterial adhesion. For example, the adhesion of *Pseudomonas aeruginosa* and *Staphylococcus aureus* on unpolished stainless-steel samples decreased significantly when S_a_ was approximately doubled [69]. The scenario for natural surfaces is less clear. Thus, moving forward, shell surface properties need to be examined at a considerably smaller scale, to obtain a better holistic view of the shell microenvironment. A worthwhile avenue for further exploration would be to determine whether S_a_ and its potential associated topography differ around the pore openings, as the pores are the main entry route of microbes through the shell, and reducing microbial adhesion immediately surrounding these could have significant positive benefits.

### 4.2. Comparisons of wettability between brood-parasitic species and hosts

We hypothesized that brood-parasitic species would have less wettable (more hydrophobic) eggshell surfaces to decrease their vulnerability to microbial attack, as nests containing eggs of brood-parasitic species have been shown to have higher infection loads, at least in some brood-parasitic systems [31,32]. We found no consistent evidence for this hypothesis, suggesting that more important selection pressures on eggshell CA, such as thermoregulation and crypsis (e.g. Attard *et al.* [38]), are shared by parasitic and non-parasitic species. CA affects thermoregulation because adherence of water droplets to the surface of the shell increases heat loss through evaporation [70,71]. Given that maintaining a constant temperature is essential for optimal embryo development [72,73], the CA of parasitic eggshells might be constrained by thermal factors rather than adapted to microbial defence or other features of a parasitic lifestyle (e.g. shell thickness). Such factors could be potential mechanisms underlying our alternative hypothesis, which was that the eggshells' CA of brood-parasitic species would match that of their host eggs due to the requirements of the shared nest environment. However, this was also not supported, as CA was no more similar between brood-parasitic species and their hosts than between brood-parasitic species and randomly assigned non-hosts species. The phylogenetic signal for CA was relatively low (*H^2^* = 0.03), suggesting that CA is driven more by ecological traits rather than relatedness, making it surprising that CA was not convergent between brood-parasitic species and their hosts, given their shared nest environment.

### 4.3. Comparisons of calcium carbonate content between eggs of brood-parasitic species and hosts

We hypothesized that the eggshells of brood-parasitic species should have proportionally greater CaCO_3_ content to support rapid bone development and potentially counter against host puncture ejection. We did not find an *overall* significant difference in the proportion of CaCO_3_ in parasitic eggshells compared with those of non-parasitic species. However, there was significant variation among parasitic species in the CaCO_3_ content of their eggshells. In particular, eggshells of Horsfield's bronze-cuckoos (*Chalcites basalis*) and little bronze-cuckoos (*Chalcites minutillus*) had comparatively lower percentages of CaCO_3_ than the other parasitic species and most of the host species. It is not immediately clear what may be driving these differences, as both of the bronze-cuckoo species generally exhibit similar parasitic behaviour to common cuckoos, in terms of rapid laying and ejection of the host eggs and young by the parasite hatchling. The lower percentages of CaCO_3_ in these two species may be linked to diet and the availability of calcium during egg production, but further work is required to confirm this.

One possible mechanism that brood-parasitic species may use to supplement their CaCO_3_ intake to produce comparatively thicker eggshells is the ingestion of host eggs. Ingestion of host eggs is common in many parasitic species (e.g. common cuckoos and Horsfield's bronze-cuckoos) and may be sufficient to compensate for the parasite's increased CaCO_3_ demands due to producing more eggs than hosts. However, this does not explain how those species which do not ingest their hosts' eggs (e.g. greater and lesser honeyguides) sequester sufficient CaCO_3_ for producing eggshells with comparatively high CaCO_3_ content. Again, further study with a greater phylogenetic spread of parasitic species is required to determine which factors (e.g. diet, location and host specificity) may dictate CaCO_3_ content of parasitic eggshells. An alternative explanation may lie in the longer laying intervals exhibited by many parasitic species (e.g. common cuckoos, greater and lesser honeyguides) compared with host species (48 h between eggs rather than 24 h [74]). Such brood-parasitic species may have more time to acquire additional calcium from their diet, and/or (in species that do ingest host eggs) to digest the calcium from the host egg they consumed when they last laid an egg of their own.

### 4.4. Conclusions and future research directions

We found that eggshell S_a_ was shared between parasitic species and their hosts, suggestive of convergent adaptation to shared nest environments. However, no consistent differences between parasites and hosts were observed in this or any other structural trait measured. Thus, although the eggshells of brood-parasitic species show many functional differences from non-parasitic species in their hardness, shape and microbial defence, the mechanisms underlying these do not seem to be due to the structures investigated here. The present study focused primarily on the outer structural components of the eggshell; future research might consider exploring other components of the eggshell, such as the chemical properties of the eggshells of brood-parasitic species, particularly the outer shell surface (cuticle), to clarify whether and how parasitic eggshells function to enable development in host nests. Moreover, future research should consider which kinds of ecological variation among parasitic species (e.g. whether hosts are grasp or puncture ejectors, whether the parasitic female eats the host egg upon removal) could drive heterogeneity in the eggshell properties of brood-parasitic species, obscuring any overall difference between brood-parasitic species and hosts. When brood-parasitic species have multiple specialized host-races (gentes), analyses should ideally match parasitic samples to those of the appropriate host species for a far greater range of gentes than in the present study. This would be particularly worthwhile in common cuckoos, given their wide geographical range and number of hosts [14]. Finally, our assumption that parasitized host nests exhibit higher bacterial loads than unparasitized nests needs to be validated across a larger set of species and host–parasite systems to better understand the ecological selection pressures acting on brood-parasitic eggs.

## Ethics

All eggshell fragments were either gathered under licence (DNPW/8/27/1) or were historic eggs available at The Natural History Museum Tring or The Western Foundation of Vertebrate Zoology. We thank the Department of National Parks and Wildlife in Zambia for support and permits (permit number DNPW/8/27/1).

## Data Availability

All data are available as an electronic supplementary material file [75].
